# Cardiopulmonary Exercise Testing in Preoperative Assessment: From Exercise Physiology to Personalized Perioperative Care

**DOI:** 10.3390/jcdd13100526

**Published:** 2026-10-20

**Authors:** Carlo Vignati, Piergiuseppe Agostoni, Beatrice Pezzuto

**Affiliations:** 1 Department of Critical Cardiology and Rehabilitation, Centro Cardiologico Monzino, IRCCS, 20138 Milan, Italy; 2 Department of Clinical Sciences and Community Health–Cardiovascular Section, University of Milan, 20122 Milan, Italy

**Keywords:** cardiopulmonary exercise testing, preoperative assessment, perioperative medicine, prehabilitation, exercise physiology, personalized perioperative care

## Abstract

Major surgery imposes physiological stress requiring coordinated adaptation of the cardiovascular, respiratory, hematological, and musculoskeletal systems. Conventional preoperative assessment remains essential but may underestimate heterogeneity in physiological reserve because resting measurements incompletely capture the response to increasing metabolic demand. Cardiopulmonary exercise testing (CPET) provides an integrated assessment of oxygen transport and utilization, linking pulmonary ventilation and gas exchange to cardiovascular performance and peripheral oxygen extraction. This narrative review examines the role of CPET in preoperative assessment, from prognostic testing to physiological phenotyping and personalized perioperative care. We summarize the physiological basis of key variables, including peak oxygen uptake, anaerobic threshold, oxygen pulse, ventilatory efficiency, end-tidal carbon dioxide, breathing reserve, and exercise kinetics, and discuss how integrated interpretation can identify patterns suggestive of haemodynamic, ventilatory, pulmonary vascular, or peripheral limitations. Evidence across major surgical populations is critically appraised, with attention to isolated thresholds and the distinction between prognostic association and clinical effectiveness. The current data support selective rather than universal use of CPET, particularly when functional capacity is uncertain or physiological information may alter management. We propose a phenotype-oriented framework in which CPET findings identify potentially modifiable impairments and inform cardiovascular optimization, respiratory treatment, nutritional support, exercise training, and prehabilitation. Emerging approaches combining complex CPET, non-invasive haemodynamic assessment, wearable technologies, and artificial intelligence may enable personalized profiling, although prospective validation remains necessary. CPET should therefore be considered not simply a risk-prediction tool, but an in vivo interrogation of physiological reserve that can contribute to mechanism-oriented perioperative assessment.

## 1. Introduction

Major surgery induces profound cardiovascular, respiratory, metabolic, inflammatory and neuroendocrine responses that challenge the organism’s ability to maintain adequate oxygen delivery and tissue homeostasis. Despite major advances in surgical techniques, anesthesia and perioperative care, cardiopulmonary complications remain a leading cause of postoperative morbidity, prolonged hospitalization and mortality, particularly among multimorbidity older patients. Consequently, accurate preoperative identification of patients with limited physiological reserve has become a central objective of contemporary perioperative medicine [1,2,3,4,5].

Current international guidelines recommend a stepwise preoperative assessment integrating clinical risk indices, procedure-related risk, functional capacity and selective use of additional investigations. However, this approach relies largely on resting variables that incompletely reflect the integrated response required to withstand surgical stress. Patients with similar comorbidity profiles may experience different postoperative outcomes, highlighting the limitations of conventional disease-based risk stratification [1,2]. Attention has therefore shifted towards physiological reserve, defined as the integrated capacity of the cardiovascular, respiratory, hematological and musculoskeletal systems to augment oxygen delivery and utilization during stress. Because major surgery represents a major physiological challenge, objective assessment of exercise performance provides an opportunity to quantify this reserve before surgery [6,7,8].

Cardiopulmonary exercise testing (CPET) is the reference standard for integrated assessment of exercise physiology. By simultaneously evaluating pulmonary ventilation, gas exchange, cardiovascular reserve and peripheral oxygen utilization during incremental exercise, CPET provides mechanistic information that complements resting investigations and may reveal interactions that are not apparent when individual components are assessed separately. Consequently, CPET is increasingly incorporated into perioperative pathways for selected patients undergoing major surgery, particularly when functional capacity is uncertain or the physiological information is expected to influence clinical decision-making [1,9,10,11].

Nevertheless, important challenges remain. Evidence supporting routine CPET across different surgical populations is heterogeneous, prognostic thresholds vary considerably among studies, and its incremental value beyond established clinical risk models remains debated. The Measurement of Exercise Tolerance before Surgery (METS) study, for example, questioned the predictive performance of some traditional CPET variables while simultaneously reinforcing the importance of the objective assessment of functional capacity, illustrating the complexity of translating physiological measurements into clinical decision-making [12,13].

Recent evidence reinforces the need to avoid treating CPET as a binary gatekeeping test. In contemporary thoracic and complex colorectal cohorts, individual variables often showed limited discrimination in the overall population, whereas clinically relevant associations emerged in selected high-risk subgroups or when CPET was integrated with pulmonary function, operative complexity and other clinical predictors [14,15].

At the same time, perioperative medicine is evolving beyond conventional risk prediction, historically based on procedure-related risk and the patient’s clinical condition, as exemplified by the Revised Cardiac Risk Index [16]. Increasing emphasis is now placed on identifying potentially modifiable physiological impairments that can be targeted through prehabilitation, nutritional optimization, cardiovascular management and personalized perioperative pathways. Within this framework, CPET may be regarded not only as a prognostic test but also as a physiological assessment capable of characterizing patterns of impaired reserve and supporting precision perioperative care [7,10].

In this review, we examine the evolving role of CPET in preoperative evaluation. We discuss physiological reserve and the basis of exercise testing, critically review the evidence supporting CPET in surgical patients, examine the limitations of threshold-based interpretation, and propose a phenotype-oriented framework for personalized perioperative optimization. Finally, we discuss digital physiology and artificial intelligence as tools to enhance—rather than replace—physiological reasoning. The primary focus is preoperative CPET; repeat testing after targeted prehabilitation is considered when it may alter management, whereas routine postoperative or longitudinal CPET remains beyond the principal scope of this review.

## 2. The Evolution of Perioperative Risk Assessment: From Disease Burden to Physiological Reserve

During the 1990s, functional capacity emerged as an additional prognostic domain. The early studies of Older and colleagues showed that reduced aerobic capacity measured by cardiopulmonary exercise testing (CPET) was associated with postoperative mortality, while the work in thoracic surgery established exercise testing as part of the physiological evaluation of candidates for lung resection [6,17].

Preoperative assessment initially developed as a disease-centred process, aimed primarily at identifying active cardiovascular disease, major comorbidity and procedure-related risk. Clinical indices and selective cardiac testing were subsequently incorporated into stepwise algorithms, an approach that remains central to current European and American guidelines [1,2].

The anaerobic threshold (AT) subsequently became particularly influential in perioperative assessment because it provided a submaximal marker associated with surgical risk. Experimental work by Koike and colleagues showed that reducing oxygen transport lowered oxygen uptake above, but not below, the metabolic acidosis threshold, supporting AT as the transition to an exercise domain in which adequate oxygen delivery becomes increasingly important [18].

A landmark prospective cohort of 548 older or cardiopulmonary-compromised patients undergoing major intra-abdominal surgery operationalized this concept by combining the anaerobic threshold, exercise-induced ischaemia, ventilatory abnormalities and procedure-related stress to allocate patients to intensive care, high-dependency care or ward management. No cardiopulmonary deaths occurred among patients selected forward care, although the observational design and the simultaneous change in postoperative management preclude attributing improved outcomes specifically to CPET-guided triage [6]. Subsequent historical reviews framed this work as a shift from screening predominantly for myocardial ischaemia toward quantifying the capacity to augment oxygen delivery in response to surgical stress [19,20].

In the following decade, perioperative assessment broadened from the detection of single-organ disease to the objective evaluation of the integrated response to stress. This development was driven by the limited ability of resting investigations and unaided clinical judgement to characterize functional limitation, particularly in older patients with multimorbidity or frailty [21].

More recent pathways have incorporated objective functional assessment into shared decision-making, postoperative triage and prehabilitation. At the same time, large prospective studies have shown that self-reported exercise capacity and isolated CPET thresholds do not consistently add predictive value across all surgical populations, challenging the use of CPET as a universal gatekeeping test [12,15,22].

The contemporary role of preoperative evaluation is therefore broader than estimating the probability of complications. Its purpose is to identify the mechanisms that limit adaptation to surgical stress and to determine whether those limitations are modifiable through earlier optimization, rehabilitation, nutritional support or changes in perioperative planning [23].

## 3. Physiological Reserve and the Oxygen Transport Pathway

Major surgery produces a coordinated neuroendocrine, inflammatory and metabolic response that increases oxygen demand while altering cardiovascular, respiratory and microcirculatory function. Tolerance of this stress depends on the ability of the organism to increase oxygen delivery and utilization while maintaining cellular energy production [3,4].

The oxygen transport pathway provides the physiological framework for this response. Oxygen must pass from ambient air to the mitochondria through pulmonary ventilation, alveolar-capillary gas exchange, blood transport and systemic circulation, followed by delivery, extraction and utilization within skeletal muscle [24]. Impairment at more than one level is common in patients undergoing major surgery.

Pulmonary ventilation must increase in proportion to metabolic demand, while effective alveolar ventilation and ventilation–perfusion matching maintain arterial oxygenation and carbon dioxide elimination. Abnormal respiratory mechanics, increased dead space or pulmonary vascular disease may therefore reduce ventilatory efficiency and limit exercise tolerance.

Systemic oxygen delivery is determined principally by cardiac output and arterial oxygen content. Cardiac output reflects the interaction between heart rate and stroke volume, whereas arterial oxygen content depends largely on hemoglobin concentration and saturation. Anemia, impaired chronotropic response, ventricular dysfunction, either systolic or diastolic, or valvular disease can each reduce oxygen delivery during stress.

At the peripheral level, blood flow must be distributed to active tissues and oxygen extracted across the microcirculation. Skeletal-muscle capillary density, mitochondrial function, muscle mass and quality, fibre composition, nutritional status and conditioning influence peripheral oxygen extraction and oxidative metabolism. Sarcopenia, cachexia, deconditioning and impaired microvascular or mitochondrial function may therefore reduce exercise capacity even when central responses appear relatively preserved [25,26,27].

Conventional resting investigations describe individual components of this pathway but do not directly test their integrated behaviour during increasing metabolic demand. CPET complements echocardiography, pulmonary function testing, biomarkers and clinical risk indices by examining the dynamic response of the complete oxygen transport system.

Peak VO_2_ reflects overall system performance, whereas ventilatory efficiency, oxygen pulse and gas-exchange variables provide complementary physiological windows into central, pulmonary and peripheral responses.

## 4. Cardiopulmonary Exercise Testing: Interrogating the Oxygen Transport Pathway

**Figure 1:**
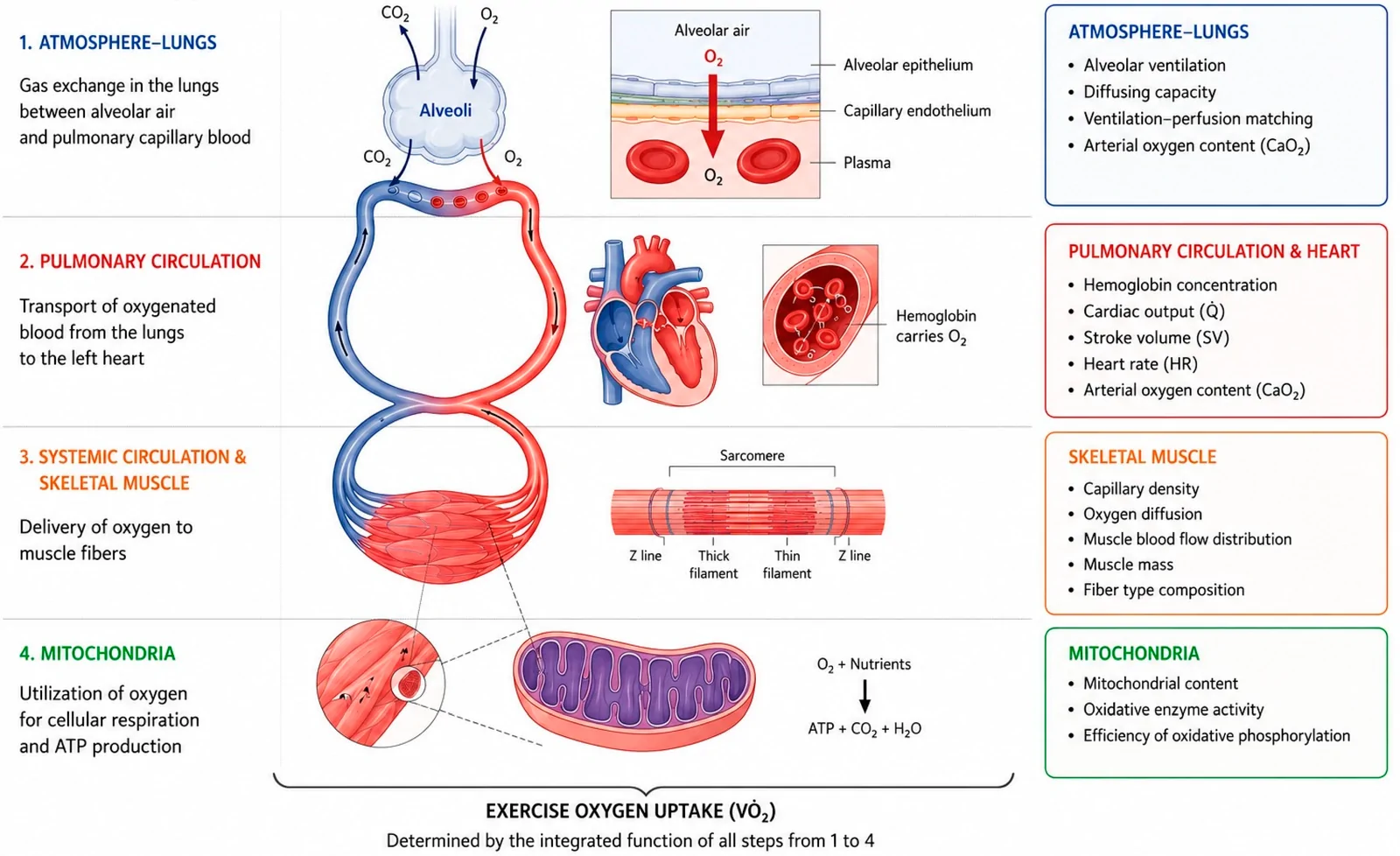
Integrated oxygen transport pathway and the principal physiological windows provided by CPET. The schematic emphasizes the sequential yet interdependent contributions of pulmonary ventilation and gas exchange, pulmonary and systemic circulation, hemoglobin-mediated oxygen carriage, skeletal muscle oxygen delivery and peripheral extraction, and mitochondrial utilization. Consistent with the Wasserman “gears” concept, dysfunction at any one level may become the dominant limiting step while abnormalities at other levels remain less apparent; integrated CPET interpretation is therefore required. ChatGPT (OpenAI) was used to create this figure from scratch based on the author’s instructions. The author reviewed and approved the figure and takes full responsibility for its content.

Cardiopulmonary exercise testing (CPET) is the reference standard for the integrated evaluation of exercise physiology. Unlike conventional investigations, which assess individual organ function under resting conditions, CPET examines the integrated response of ventilation, pulmonary gas exchange, circulation and peripheral oxygen utilization during increasing metabolic demand (Figure 1). This is consistent with the classical Wasserman “gears” concept, in which exercise capacity emerges from coordinated operation of multiple physiological components; failure of one component may become the dominant limiting step while abnormalities elsewhere remain less apparent [25].

### 4.1. The Fick Principle: Integrating Oxygen Delivery and Utilization

The physiological foundation of CPET is the Fick principle:

VO_2_ = Cardiac Output × (CaO_2_ − CvO_2_)

This equation emphasizes that oxygen uptake reflects the interaction between central oxygen delivery and peripheral oxygen extraction. Cardiac output depends on heart rate and stroke volume, whereas the arteriovenous oxygen-content difference is influenced by hemoglobin concentration, pulmonary gas exchange, skeletal muscle perfusion and mitochondrial oxidative capacity. Peak oxygen uptake therefore represents the integrated performance of the entire oxygen transport pathway rather than a measure of cardiovascular function alone [25,26,27,28,29]. Importantly, the Fick formulation does not explicitly represent the distribution of blood flow to the working muscle, which may change substantially during exercise and after therapeutic interventions. Recent work has therefore emphasized that peak VO_2_ should be interpreted together with cardiac output and arteriovenous oxygen difference, while complex CPET can add non-invasive haemodynamic and peripheral measurements when the mechanism of limitation remains uncertain [30,31,32,33,34]. Identical reductions in peak VO_2_ may therefore arise from impaired stroke-volume augmentation, chronotropic incompetence, pulmonary vascular dysfunction, anemia, skeletal-muscle deconditioning or mitochondrial dysfunction [9,25,27].

### 4.2. CPET Variables as Physiological Windows

Peak VO_2_ provides a global measure of integrated aerobic capacity. The anaerobic threshold identifies the transition towards increasing reliance on anaerobic metabolism and provides a submaximal marker of exercise capacity. The VE/VCO_2_ slope reflects ventilatory efficiency and is influenced by ventilation–perfusion matching, pulmonary vascular function, dead-space ventilation and, in heart failure, circulatory abnormalities. The oxygen pulse reflects VO_2_/heart rate and is influenced by stroke volume and peripheral oxygen extraction; its trajectory is therefore more informative than an isolated value. PETCO_2_ and PETO_2_ provide complementary information on ventilation, gas exchange and circulatory–pulmonary interaction. Breathing reserve estimates ventilatory headroom at peak exercise. These variables should be interpreted together because no single index captures the full physiological response [25,35,36].

The physiological rationale for perioperative CPET is that surgery, like exercise, increases oxygen demand and exposes limitations in the integrated oxygen transport pathway that may remain unapparent at rest. Anaerobic-threshold values near 11 mL/kg/min and other commonly used cut-offs are therefore risk markers rather than physiological boundaries, and their meaning depends on the clinical and testing context [7,17,37,38,39,40,41].

In lung-resection candidates, resting PETCO_2_ may also provide a pragmatic window on ventilatory inefficiency: in a prospective multicentre cohort, lower resting PETCO_2_ and a higher VE/VCO_2_ slope were independently associated with postoperative pulmonary complications and showed similar model discrimination [42]. Importantly, the oxygen pulse should be interpreted as VO_2_/heart rate rather than as a direct measure of stroke volume, because it is also influenced by the arteriovenous oxygen-content difference. Its trajectory is therefore most informative when considered together with heart-rate response, blood-pressure response, other CPET variables and, when indicated, direct or non-invasive haemodynamic measurements [25,35,36].

Viewed individually, these variables offer only partial physiological information. Their principal value emerges when interpreted together, as in the traditional Wasserman nine-panel approach, which helps identify the predominant pattern limiting exercise performance. Interpretation should proceed across panels to determine whether the pattern is suggestive of haemodynamic, ventilatory-mechanical, pulmonary vascular/gas-exchange, peripheral, or mixed limitation; CPET findings remain suggestive rather than independently diagnostic [25,35,36,43,44].

**Figure 2:**
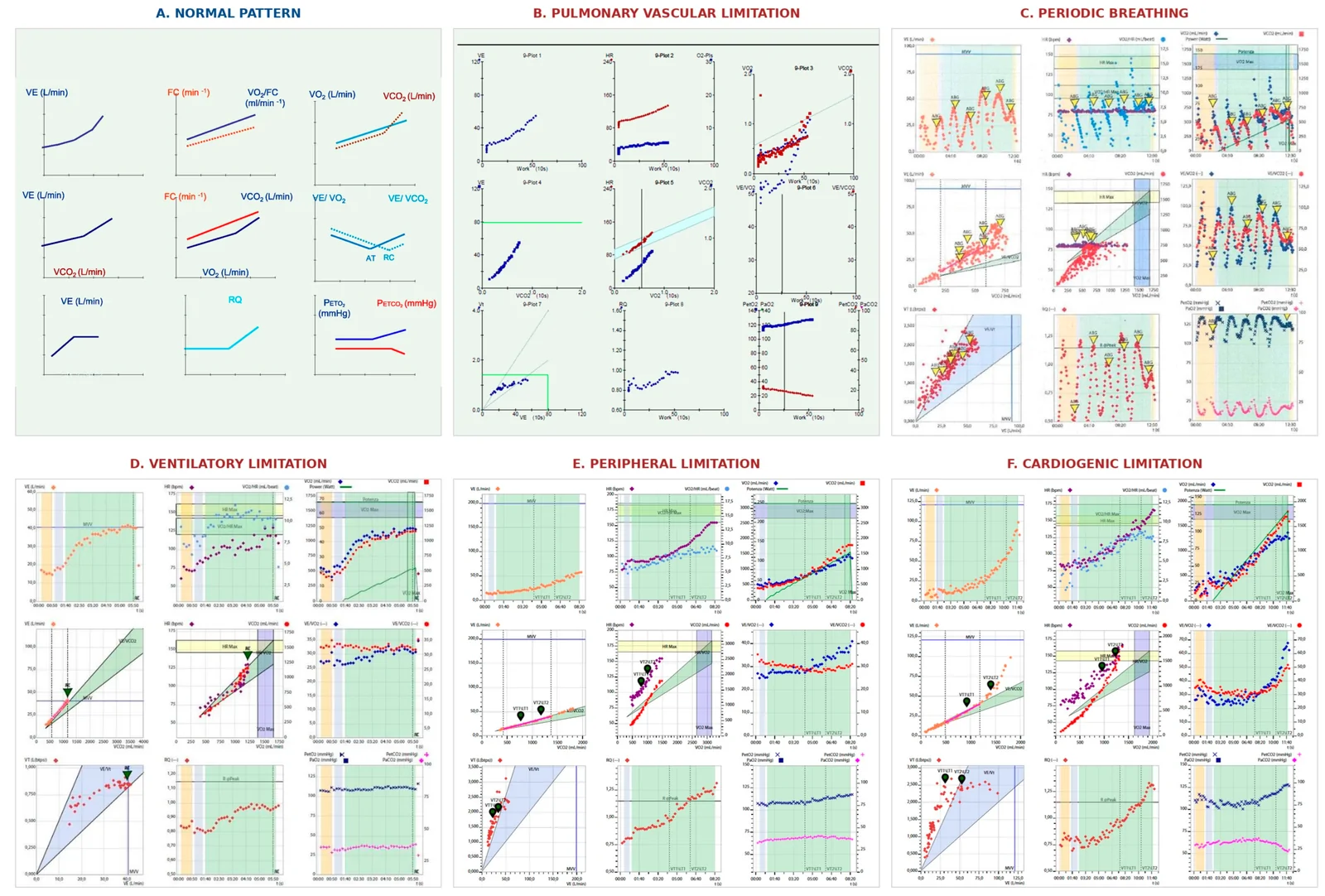
Schematic nine-panel framework for integrated CPET interpretation (original didactic representation). The figure is intended to illustrate pattern recognition rather than establish diagnostic phenotypes. The main signals are global aerobic capacity and kinetics (VO_2_/work rate); cardiovascular response (heart-rate and O_2_-pulse trajectories); ventilatory response and efficiency (VE, VE/VCO_2_); gas exchange (PETCO_2_, PETO_2_, oxygen saturation); and, when available, ventilatory mechanics. Panel C (periodic breathing) represents an exercise-ventilatory pattern with prognostic relevance in selected populations rather than a stand-alone mechanism of exercise limitation. Mixed patterns are common and all panels should be interpreted together and in clinical context.

Typical pattern recognition is intentionally simplified (Figure 2): a haemodynamic pattern may show reduced or flattening O_2_-pulse and/or VO_2_ kinetics with a relatively appropriate ventilatory response; a pulmonary vascular/gas-exchange pattern may combine ventilatory inefficiency, low PETCO_2_ and, in some cases, desaturation; a ventilatory-mechanical pattern may show reduced breathing reserve with expiratory flow limitation, reduced inspiratory capacity or dynamic hyperinflation; and a peripheral/deconditioning pattern may show reduced aerobic capacity and early metabolic limitation despite comparatively preserved central responses. Panel C is a prognostic exercise-ventilatory pattern rather than a specific mechanism. These patterns are not mutually exclusive.

### 4.3. Practical Considerations and Current Limitations

Despite its unique physiological information, CPET requires specialized equipment, experienced personnel and standardized protocols, limiting its availability outside dedicated centres. Heterogeneity among studies regarding exercise protocols, interpretation criteria and outcome definitions also contributes to variability in proposed prognostic thresholds [1,11,12,13]. These limitations favour selective, clinically question-driven use of CPET.

For these reasons, simpler functional assessments—including the six-minute walk test (6MWT), stair-climbing tests and the 30 s chair-stand test—have gained increasing attention as pragmatic tools for estimating functional capacity. However, while these assessments may identify reduced physical performance, they cannot determine whether exercise limitation results from impaired cardiac reserve, ventilatory inefficiency or peripheral oxygen utilization. They should therefore be regarded as complementary screening tools rather than substitutes for CPET when detailed physiological characterization is required [45]. Moreover, the 6MWT is generally considered a submaximal field test [43,46], although its physiological load varies between individuals and may become high or even near-maximal in some patients with severe disease [44,46,47].

The stair-climbing literature illustrates both the clinical usefulness and the physiological limitations of field tests. Inability to climb two flights of stairs has been associated with postoperative cardiopulmonary complications and, in prospective thoracic cohorts, with substantially higher mortality. However, stair climbing is brief, non-standardized and strongly influenced by musculoskeletal performance and anaerobic energy contribution; it may therefore either overestimate or underestimate sustained aerobic reserve. Accordingly, stair climbing is best used as an initial screening tool, whereas CPET remains preferable when the clinical question concerns the aerobic capacity required to tolerate prolonged major surgical stress [17,48].

Brief submaximal protocols may broaden access, but their role remains investigational. In a small prospective study of older higher-risk patients, a gas-exchange-derived pulmonary capacitance slope was associated with early postoperative morbidity and length of stay, outperforming conventional peak values and questionnaire estimates; external validation is required before routine implementation [49].

## 5. Clinical Evidence: What Does CPET Really Add to Perioperative Assessment?

The incorporation of CPET into perioperative assessment has been driven by the association between functional capacity and postoperative outcome. The key clinical question is whether CPET adds useful information to conventional risk assessment and identifies potentially modifiable limitations [1,6,7,8].

Most evidence supporting CPET derives from prospective and retrospective observational studies, whereas randomized trials evaluating CPET-guided perioperative strategies remain limited. Current evidence is therefore stronger for prognostic association than for the impact of CPET-guided interventions on postoperative outcomes. This distinction is essential when moving from physiological phenotyping to treatment decisions [1,2,12].

### 5.1. Evidence Across Surgical Specialties

The evidence supporting CPET is remarkably consistent across diverse surgical populations. In thoracic surgery, reduced peak oxygen uptake has repeatedly been associated with increased postoperative pulmonary complications, prolonged hospitalization and mortality, leading international thoracic guidelines to incorporate CPET into the operability assessment for selected patients with impaired pulmonary reserve [17,50,51].

The updated ERS/ESTS guideline adopts a tiered rather than universal approach: low-technology exercise testing is suggested as an initial functional screen, with formal CPET reserved principally for patients with impaired pulmonary function or suboptimal field-test performance. The panel did not endorse a single universal CPET parameter or cut-off, although a peak VO_2_ below 12 mL/kg/min or a VE/VCO_2_ slope above 40 may indicate high surgical risk [50].

The distinction between prognostic association and clinical effectiveness is important. A systematic review and meta-analysis of preoperative stress testing found that the available comparative evidence was insufficient to establish that testing itself reduces 30-day mortality after non-cardiac surgery. This does not negate the prognostic information provided by CPET, but it emphasizes that improved outcomes depend on whether test findings lead to effective changes in surgical planning, optimization of patient’s condition or postoperative care [8].

Recent evidence in thoracic surgery illustrates both the potential and the limitations of single-variable interpretation. A large prospective NSCLC cohort found that a VE/VCO_2_ slope of at least 31 was associated with perioperative morbidity and worse relapse-free and overall survival [52]. Conversely, a later single-centre cohort found poor stand-alone discrimination for peak VO_2_, anaerobic threshold and VE/VCO_2_ slope, despite an independent association between ventilatory inefficiency and complications [14]. These findings support population-specific and multivariable interpretation rather than universal cut-offs. The VE/VCO_2_ slope also varies with age and sex. More generally, when validated reference equations are available, expressing peak VO_2_ and other suitable variables as a percentage of predicted values can contextualize relative functional capacity; predicted values should complement, not replace, absolute measures and clinical context [35,36,50].

A contemporary multicentre secondary analysis of 497 lung-resection candidates further challenged the incremental predictive role of CPET. Across five machine-learning approaches, adding CPET variables to clinical data, arterial blood gases and pulmonary function tests did not significantly improve prediction of pulmonary or cardiovascular complications, including among patients meeting ACCP or ERS/ESTS criteria for testing. Nevertheless, VE/VCO_2_ slope, sex- and age-adjusted peak VO_2_ and resting PETCO_2_ remained among the more influential predictors, suggesting that useful physiological information may be diluted when CPET is reduced to a small set of uncontextualized predictors [22,53].

Similarly, observational studies in major abdominal surgery have shown that lower anaerobic threshold and peak VO_2_ are associated with increased postoperative morbidity after colorectal, hepatopancreatobiliary and upper gastrointestinal procedures [6,8,54]. Comparable findings have been reported in vascular surgery, where impaired aerobic capacity identifies patients with limited cardiovascular reserve undergoing abdominal aortic aneurysm repair or major lower-limb revascularization [2,8].

In oesophageal and gastric cancer surgery, a prospective cohort reported a strong association between a low anaerobic threshold and cardiovascular complications, with an AT below 9.5 mL/kg/min providing a high negative predictive value for major cardiac events [54]. In complex rectal cancer surgery, however, AT and peak VO_2_ were not associated with outcomes across the full cohort but showed exploratory discrimination in the total pelvic exenteration subgroup [15].

In oesophageal cancer, simpler assessments may also add clinically useful information when CPET is unavailable or impractical. A 2026 multicentre observational cohort found that the 30 s chair-stand test was more frequently completed than the 6MWT and improved reclassification and discrimination for postoperative pulmonary complications when added to established clinical risk factors. The proposed threshold of fewer than 15 repetitions was exploratory and requires external validation, but the findings support a tiered pathway in which simple tests identify patients who may benefit from more comprehensive physiological assessment [45].

Although these observations consistently support the prognostic value of reduced exercise capacity, substantial heterogeneity exists regarding optimal CPET variables, proposed cut-off values and outcome definitions. Differences in surgical complexity, patient characteristics, perioperative management and enhanced recovery protocols likely explain much of this variability, making universal prognostic thresholds difficult to define [1,7].

Rather than representing a limitation of CPET itself, this heterogeneity reflects the biological complexity of contemporary surgical populations. Exercise intolerance is influenced not only by cardiovascular reserve but also by ventilatory mechanics, pulmonary vascular function, frailty, sarcopenia, nutritional status, anemia, chronic inflammation and cancer-related physiological impairment. Threshold-only interpretation may therefore oversimplify perioperative physiology [1,7].

### 5.2. The METS Study: From Prediction to Physiology

The METS study represents a landmark in contemporary perioperative medicine [12,13]. Beyond evaluating CPET, the study challenged the reliability of subjective assessment of functional capacity by demonstrating poor agreement between clinician-estimated exercise tolerance and objectively measured peak VO_2_.

Equally important, the METS highlighted that traditional CPET variables provided only modest incremental prediction for some postoperative outcomes, whereas biomarkers such as NT-proBNP demonstrated stronger prognostic performance for cardiovascular complications. These findings have sometimes been interpreted as questioning the clinical utility of CPET. However, such an interpretation overlooks the distinct objectives of risk prediction and physiological assessment.

METS should therefore not be interpreted as evidence that CPET lacks perioperative value. Rather, the study showed that clinician-estimated functional capacity was inaccurate, that the Duke Activity Status Index (DASI) and NT-proBNP were useful for selected cardiovascular outcomes, and that lower peak VO_2_ was associated with broader postoperative complications. Its central implication is that test selection should follow the clinical question: questionnaires and biomarkers may support risk stratification, whereas CPET offers objective physiological characterization [12].

Resting pulmonary function testing provides complementary information, particularly in thoracic surgery. Spirometry characterizes ventilatory mechanics through measures such as FEV1 and FVC, while diffusing capacity (DLCO) reflects alveolar-capillary gas transfer; predicted postoperative FEV1 and DLCO remain central to physiological assessment before lung resection [17,50]. These measurements, however, assess specific components of the respiratory function predominantly at rest, whereas CPET evaluates the integrated cardiovascular, ventilatory and peripheral response to increasing metabolic demand.

Prediction models aim to maximize discrimination for postoperative events, whereas CPET seeks to characterize the patterns and mechanisms associated with impaired physiological reserve. These objectives are complementary. METS therefore supports moving beyond threshold-based interpretation while retaining the role of simpler functional measures, biomarkers and conventional assessment.

### 5.3. CPET in Current Guidelines

Current European and North American guidelines recognize CPET as the most comprehensive objective method for evaluating the functional capacity in selected patients undergoing major surgery [1,2]. Neither guideline recommends routine CPET for all surgical candidates; instead, CPET is advised when functional capacity cannot be reliably estimated or when the physiological information is expected to influence management [1,2].

This selective approach acknowledges both the strengths and practical limitations of CPET, including its requirement for specialized equipment, trained personnel and expert interpretation.

Recent interest in simpler functional assessments—including the 6MWT, stair-climbing tests and the 30 s chair-stand test—reflects the need for pragmatic alternatives in centres where CPET is unavailable [45]. Although these tests may identify reduced physical performance, they provide limited mechanistic insight and should therefore be considered complementary screening tools rather than substitutes for comprehensive physiological assessment.

### 5.4. Critical Appraisal and Remaining Knowledge Gaps

Taken together, the evidence consistently associates reduced exercise capacity with adverse postoperative outcomes, while the incremental predictive value of individual CPET variables varies across populations. The effectiveness of phenotype-guided management remains incompletely established.

A systematic review of 36 studies in non-cardiopulmonary surgery found that CPET-derived thresholds generally had stronger negative than positive predictive value, while prediction models were infrequently validated and often at risk of bias or overfitting [55]. These findings argue against excluding patients from surgery on the basis of an isolated abnormal value.

Most studies have focused on prognostic cut-offs rather than phenotype-guided interventions; whether physiology-informed optimization improves clinically meaningful outcomes remains uncertain [7,10,56].

## 6. From Risk Stratification to Physiological Phenotyping: A New Framework for Precision Perioperative Medicine

Physiological phenotyping integrates peak VO_2_, anaerobic threshold, oxygen pulse, VE/VCO_2_ slope, PETCO_2_, breathing reserve, oxygen saturation and exercise kinetics to identify patterns suggestive of the predominant limitation.

These phenotypes are working descriptions rather than diagnostic categories; mixed patterns are common, and definitive diagnosis requires appropriate clinical, imaging, laboratory or pulmonary assessment.

The following phenotypes should therefore be viewed as a framework for clinical reasoning rather than validated treatment algorithms.

### 6.1. Central Haemodynamic Limitation

Patients with impaired stroke-volume augmentation or chronotropic incompetence may demonstrate reduced peak VO_2_ with a low or flattening oxygen-pulse trajectory, while ventilatory efficiency is relatively preserved. This pattern is suggestive of reduced cardiovascular reserve, but oxygen pulse is not a direct measure of stroke volume. Interpretation should include heart-rate and blood-pressure responses, ECG or ischemic features, symptoms and, when indicated, echocardiographic or other haemodynamic assessment [2,25,57,58,59].

### 6.2. Ventilatory and Pulmonary Vascular Limitation

Another pattern is characterized by disproportionate ventilatory inefficiency, elevated VE/VCO_2_ slope, reduced PETCO_2_ and, in selected patients, exercise-induced oxygen desaturation. These abnormalities are suggestive of impaired ventilation–perfusion matching, pulmonary vascular dysfunction or advanced chronic respiratory disease. Ventilatory mechanics deserve separate consideration: reduced breathing reserve (VEpeak/MVV) indicates limited ventilatory headroom but does not establish the cause of limitation. When relevant, interpretation should incorporate exercise flow-volume loops, serial inspiratory-capacity maneuvers, operating lung volumes and evidence of dynamic hyperinflation, together with the symptom response. In COPD and other obstructive disorders, dynamic hyperinflation and progressive encroachment on inspiratory reserve may identify clinically important mechanical constraints that are not adequately characterized by breathing reserve alone [7,50,60,61,62,63]. Recognition of this overall pattern may influence perioperative respiratory optimization, pulmonary rehabilitation, smoking cessation and postoperative ventilatory support, with appropriate confirmatory assessment [9,27].

### 6.3. Peripheral and Metabolic Limitation

Some patients exhibit early anaerobic thresholds and reduced peak VO_2_ despite relatively preserved central haemodynamics and ventilatory efficiency. This profile is suggestive of impaired skeletal muscle oxidative capacity, sarcopenia, physical inactivity, nutritional deficiency or mitochondrial dysfunction. Some contributors are potentially modifiable through exercise training, nutritional optimization and multimodal prehabilitation [10,51,64,65]. The PREHEP randomized trial provides a relevant example: in sarcopenic patients undergoing major hepatectomy, exercise plus nutritional support reduced 90-day morbidity and improved muscle-related measures, although the single-centre sample and selected population limit generalization [66].

### 6.4. Mixed Physiological Phenotypes

In clinical practice, isolated mechanisms are uncommon. Older patients undergoing major surgery frequently demonstrate combinations of cardiac dysfunction, pulmonary disease, frailty, anemia and skeletal-muscle deconditioning. CPET should therefore not be used to assign mutually exclusive phenotypes, but to estimate the relative contribution of different physiological impairments to reduced reserve [1,7,25].

This integrated interpretation represents an advantage of CPET over simpler functional assessments. Whereas walking tests or questionnaires quantify overall performance, CPET adds information about the physiological pattern accompanying reduced exercise capacity and may help determine the most appropriate additional assessment. CPET findings should nevertheless be treated as hypotheses requiring clinical confirmation rather than as stand-alone diagnoses [67].

### 6.5. Translating Physiological Phenotypes into Personalized Perioperative Care

The clinical value of physiological phenotyping lies in connecting a CPET pattern with an appropriate next clinical step rather than simply estimating the postoperative risk. A practical sequence is: CPET pattern → suspected physiological limitation → targeted additional assessment → potentially modifiable factor → perioperative action. A haemodynamic pattern may prompt cardiovascular assessment and medication optimization; a ventilatory-mechanical pattern may prompt pulmonary function testing and assessment for dynamic hyperinflation; a pulmonary vascular/gas-exchange pattern may prompt evaluation for pulmonary vascular disease or significant gas-exchange impairment; and a peripheral pattern may prompt assessment of nutrition, muscle mass and deconditioning. These links are hypothesis-generating and should be individualized to the surgical context and available evidence [10,44,56,62,64,65].

Prehabilitation deserves a defined place within this framework because physiological phenotyping may identify which component of reserve is potentially modifiable before surgery. Exercise training, respiratory-muscle training, nutritional optimization, smoking cessation and correction of selected reversible factors can be combined according to the patient’s vulnerabilities. The evidence suggests that prehabilitation can improve functional capacity and, in selected populations, reduce complications, but the programmemes and endpoints vary substantially. CPET can therefore help target and monitor prehabilitation without implying that the changes in CPET variables necessarily translate into improved surgical outcomes [35,36,68,69,70].

More direct evidence for physiology-informed intervention has emerged in thoracic surgery. In a multicentre randomized trial, patients undergoing lung resection were selected for prehabilitation on the basis of a VE/VCO_2_ slope of at least 33. A 14-day multimodal programme incorporating respiratory-muscle training, smoking cessation, nutritional support and psychological support reduced postoperative pulmonary complications, shortened hospital stay and modestly improved ventilatory efficiency [71]. This trial supports targeted intervention in a CPET-defined subgroup, although it does not establish applicability across other operations.

CPET may also have a role within Enhanced Recovery After Surgery pathways by informing shared decision-making, postoperative triage and individualized exercise prescription. In this review, however, its principal role is preoperative assessment and identification of potentially modifiable physiological limitations. Repeat CPET after targeted prehabilitation may be useful when the result could alter management or surgical timing, but routine postoperative or serial CPET is not an established component of perioperative care. Improvement in CPET variables should not automatically be equated with improved surgical outcomes [7,72].

## 7. Future Directions: Towards Precision Perioperative Physiology

Future research should validate reproducible physiological patterns and test whether patients with haemodynamic, ventilatory-mechanical, pulmonary vascular/gas-exchange or peripheral abnormalities derive differential benefit from targeted perioperative strategies [10,56,64,65].

Trials should distinguish improvement in a physiological surrogate from improvement in patient-important surgical outcomes. Exercise training can increase peak VO_2_ and physical function, but evidence that CPET-guided, phenotype-targeted prehabilitation reduces major morbidity or mortality remains limited. Future studies should predefine phenotypes, interventions and outcomes and test whether physiological changes mediate recovery. Recent randomized studies suggest the benefit may be greatest when intervention addresses a defined vulnerability, such as ventilatory inefficiency before lung resection or sarcopenia before major hepatectomy [66,71]. Anemia provides a cautionary counterpart: in the PREVENTT randomized trial, preoperative intravenous iron increased hemoglobin but did not reduce the primary outcome, illustrating that correcting a biological vulnerability does not necessarily improve perioperative outcomes [73].

Technological innovation is expanding opportunities for physiological assessment. Wearable sensors, continuous haemodynamic monitoring and digital health platforms may complement formal CPET by providing additional measurements of functional status before surgery and during recovery. Such approaches should be considered complementary to CPET rather than evidence that routine longitudinal CPET is established [22,74].

A related development is “complex CPET”, in which breath-by-breath gas exchange is combined with non-invasive cardiac-output measurement and, in selected settings, near-infrared spectroscopy. Such integration can help separate central flow limitation from impaired peripheral extraction when similar peak VO_2_ values arise from different patterns. Available techniques include inert-gas rebreathing, impedance cardiography, thoracic bioreactance and exercise echocardiography, although perioperative outcome validation remains limited [43,44,75,76]. These approaches provide a physiological rationale for adding direct haemodynamic measurements, but their routine applicability to surgical populations requires prospective validation.

Artificial intelligence (AI) and machine learning may further enhance CPET interpretation. Rather than relying only on isolated variables or predefined thresholds, AI algorithms could integrate breath-by-breath data with clinical characteristics, imaging, biomarkers and perioperative outcomes. Such multimodal approaches may improve physiological phenotyping and identify THE response patterns not captured by conventional thresholds. They should, however, augment—not replace—physiological interpretation and clinical judgement [22,74,77,78].

The recent evidence in thoracic surgery also provides an important caution: more complex algorithms do not necessarily create incremental value when predictors are redundant or population-selection criteria are poorly calibrated. Machine learning should therefore be evaluated against transparent clinical baselines, with external validation and assessment of calibration and clinical utility rather than discrimination alone [22].

Personalization may add further value, but the evidence remains preliminary. In a small single-centre randomized trial across major elective procedures, individually coached multimodal prehabilitation improved physical performance, produced measurable immune-signalling changes and was associated with fewer moderate-to-severe postoperative complications than a standard paper-based program [79]. The study was primarily designed around physiological and immunological outcomes and therefore supports biologically informed tailoring as a research direction rather than a validated CPET-guided treatment algorithm.

Future trials should determine whether CPET-guided phenotype-directed optimization improves patient-important outcomes beyond conventional risk stratification.

## 8. Conclusions

Major surgery challenges the integrated function of the cardiovascular, respiratory and musculoskeletal systems, making physiological reserve a key determinant of the postoperative outcome. Although conventional clinical assessment remains fundamental, it provides only a partial description of the patient’s ability to respond to the metabolic demands imposed by surgery.

CPET evaluates the integrated behaviour of the oxygen transport pathway during stress, providing information that cannot be obtained from resting investigations alone. While impaired exercise capacity is associated with adverse postoperative outcomes, the physiological pattern may also help identify the mechanisms associated with reduced reserve and the need for targeted additional assessment or optimization.

This perspective represents a shift from disease-based risk assessment towards mechanism-based physiological evaluation. CPET can help clinicians understand why physiological reserve appears impaired and which components of the oxygen transport pathway may be amenable to intervention, while recognizing that CPET patterns are suggestive and require clinical confirmation.

Future research should prioritize validation of physiological phenotyping, personalized perioperative optimization and integration with emerging digital technologies, while preserving the distinction between prognostic association and demonstrated treatment benefit.

In this framework, CPET should be regarded not simply as a test for perioperative risk prediction, but as an in vivo interrogation of physiological reserve that can support mechanism-oriented assessment and personalized perioperative care when integrated with the broader clinical context.

## Data Availability

No new data were created or analyzed in this study.
